# Three Years' Experience of a Novel Biosynthetic Cellulose Dressing in Burns

**DOI:** 10.1089/wound.2018.0790

**Published:** 2019-02-13

**Authors:** Matilda Karlsson, Pia Olofsson, Ingrid Steinvall, Folke Sjöberg, Johan Thorfinn, Moustafa Elmasry

**Affiliations:** ^1^ Department of Plastic Surgery, Hand Surgery, and Burns, Linköping University, Linköping, Sweden.; ^2^ Department of Clinical and Experimental Medicine, Linköping University, Linköping, Sweden.; ^3^ Department of Anaesthesiology and Intensive Care, Linköping University, Linköping, Sweden.; ^4^ Surgery Department, Suez Canal University, Ismailia, Egypt.

**Keywords:** burns, cellulose, wound healing, dressing

## Abstract

**Objective:** The use of porcine xenograft (PX) is widely spread in burn care. However, it may cause immunologic responses and other ethical and cultural considerations in different cultures. Therefore, there is a need for alternatives. The aim of this work is to test a novel biosynthetic cellulose dressing (Epiprotect^®^) on burn patients.

**Approach:** Charts from 38 patients with superficial burns (SBs) (*n* = 18) or excised burns (*n* = 20) that got biosynthetic cellulose dressing instead of PX at a national burn center during 3 years were reviewed. Time to healing, length of stay, and wound infection were extracted from the medical records.

**Results:** SBs hospitalization time was 11 days comparable to PXs reported by others. In the excised group, median duration of hospital stay was 35 days. Time to healing was 28 days. Seven wound infections were confirmed in the superficial group (39%) and 11 infections in the excised group (61%). Patients with superficial wounds reported pain relief on application.

**Innovation:** A dressing (17 × 21 cm) consisting of biosynthetic cellulose replacing PX.

**Conclusion:** Outcome of treatment of SBs or temporary coverage of excised deep burns with biosynthetic cellulose is comparable to treatment with PX. However, biosynthetic cellulose has benefits such as providing pain relief on application and ethical or cultural issues with the material is nonexistent.

**Figure:**
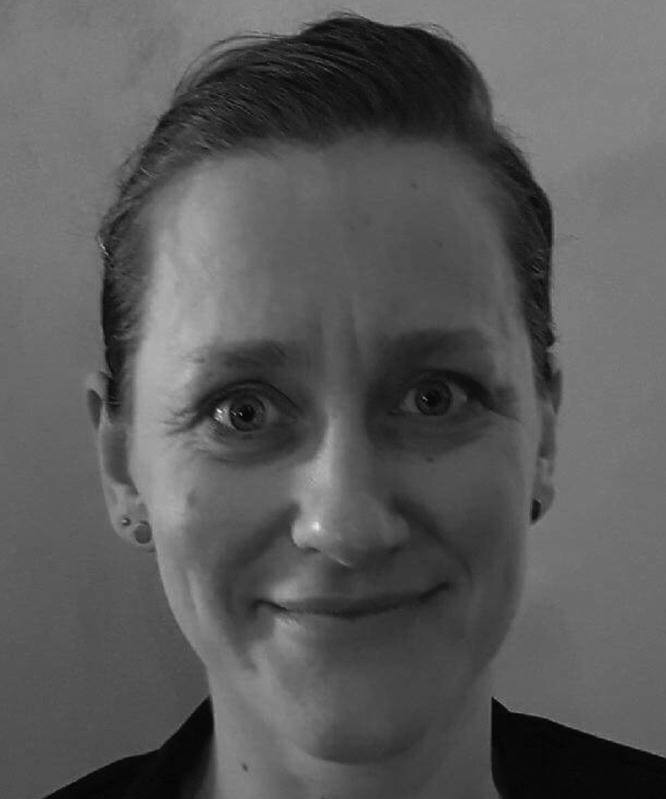
Matilda Karlsson, RN

## Introduction

In our burn center, biological membranous dressings, such as porcine xenografts (PXs), play an essential role in burn care either as temporary wound coverage or as a dressing for conservatively treated superficial wounds. In temporary covering, they serve as an effective option for wound protection in situations where the placement of autograft needs to be delayed such as unstable patient, lack of donor sites, heavily infected wounds, or awaiting demarcation. For conservatively treated wounds, the biological dressing aims to protect the wound during healing by keeping a moist wound environment and posing a barrier against infection.^1,2^ Also, it has been reported that PXs may prevent heat-, fluid- and protein loss.^3–5^

During the course of healing of superficial burns (SBs), PX dries out and peels off as the skin underneath is reepithelialized, leaving a healed surface. When using it on deep dermal or full-thickness burns, PX will usually not stick completely and peel off, but it adheres somewhat to an excised wound bed that is viable and protects the wound surface by preventing fluid evaporation and making it more difficult for bacteria to enter the wound. With these properties, some clinicians use PX as an indicator to identify where the wound bed is adequately excised and exhibits viable tissue that is ready for autografting.^6–8^ However, the use of PX is not uncontroversial because of its animal origin, and therefore, we looked for alternative materials with similar or better characteristics.

Cellulose is a large carbohydrate molecule normally present in plants. It has a dense fibrillar network that attracts water and thus has been proposed as a good candidate for a dressing. The possible toxicity of the cellulose has been evaluated *in vitro* and *in vivo* (mice) studies and has been found to be very low.^9^ Skin cells such as fibroblast, adipose stem cells, and human umbilical vein endothelial cells have shown no difference in morphology and good proliferation when cultured near or attached to cellulose.^10,11^ Therefore, it should make an ideal dressing by its properties to keep the wound moist and thus facilitate the process of epithelialization. This would come away from some of the issues with PX since cellulose in contrast to PX is a product naturally present in nature but not of animal origin, and it rises no ethical or cultural questions in production or when used.

However, previous use of traditional cellulose as a material for dressings was problematic for us. For example, it was quite compact in nature and fluids such as blood, and exudate was trapped under the membrane, which in turn promoted bacterial growth. Furthermore, traditional cellulose integrated with the wound bed in some cases, making removal very hard and painful for the patient (data not shown). Thus, in its earlier forms, we discarded it as an alternative for dressings.

With recent advances in development, biosynthetic cellulose is a new variant of cellulose. It has high purity and can be tailored to sheets.^12,13^ In its current form, it has a high water absorption capacity as well as unique mechanical properties, good permeability, and resistance to degradation. Most of these properties arise from the three-dimensional nanofibrillar network, which is similar to human skin. It is permeable to water vapor, oxygen, and carbon dioxide but at the same time forming a tight barrier to the environment, thus making it more difficult for bacterial penetration. Moreover, the material is semitransparent and flexible.^14^

## Clinical Problem Addressed

Even if there are studies showing the cost-effectiveness of PX,^3^ low humoral response, and positive impact on keratinocyte proliferation,^15–17^ PX has some limitations. It may cause unsatisfying scars even up to 8 years after healing,^18–20^ may still, according to others, have a negative impact on keratinocyte- and fibroblast growth,^21^ and also potentially transmits diseases to patients.^22^ Furthermore, the use of PX is of religious and ethical concern in some groups.^23,24^ To overcome the issues related to the use of PX, efforts have been made to identify alternative wound dressings in the recent years, of which biosynthetic cellulose is one.

The aim of this case series report is to share our early experience with the use of biosynthetic cellulose in conservatively treated SBs and surgically excised burns (EBs) in instances where PXs would have been used as the standard of care.

## Materials and Methods

This retrospective case review was conducted at a national burn center in Sweden. Approval by the Regional Ethics Review Board was taken (2015/386-31). The medical records of all patients admitted to the burn center between 2013 and 2015 were screened, and patients treated with bacterial cellulose for wound coverage of SBs or EBs were included. Demographic data for all patients such as sex and age were collected as well as information about wound type treated, application frequency, presence of wound infection, and time from first application to final wound closure (SB group only). We chose not to register time to wound closure for the EB group because the dressing was only used as temporary coverage before autotransplantation, and there are many factors other than the actions taken before transplantation that affects outcome in this kind of patients. Wounds were seen as infected if presenting positive microbial wound swabs together with an elevated plasma C-reactive protein (CRP) and/or development of systemic as well as local signs of infection. Any other adverse reactions related to the dressing, such as allergy, problem with hemostasis, and ingrowth, were not seen. Patients with wounds that were not caused by a burn injury and patients who died before first take down and/or removal of cellulose were excluded. Descriptive data are given as median (IQR 10–90) unless otherwise stated.

Application of the biosynthetic cellulose on the SBs or EB wounds was done under sterile conditions in the operating room or strictly clean conditions bedside. Two layers of paraffin gauze were applied on top of the cellulose sheet and finally covered with dry gauze.

When used as temporary cover awaiting autologous skin grafting (EB group), the dressings were removed whenever necessary to assess the wound bed. When used as conservative treatment (SB group), the dressings were left until they peeled off by themselves during healing. The dressings were checked every second to third day and, in case of suspected wound infection, the wound area was swabbed for microbial growth, then lightly cleaned with saline, and replaced with silver-containing dressings or a dialkylcarbamoyl chloride-impregnated dressing, which is the standard of care for wounds treated with PX in our unit.

## Results

**Figure 1.**
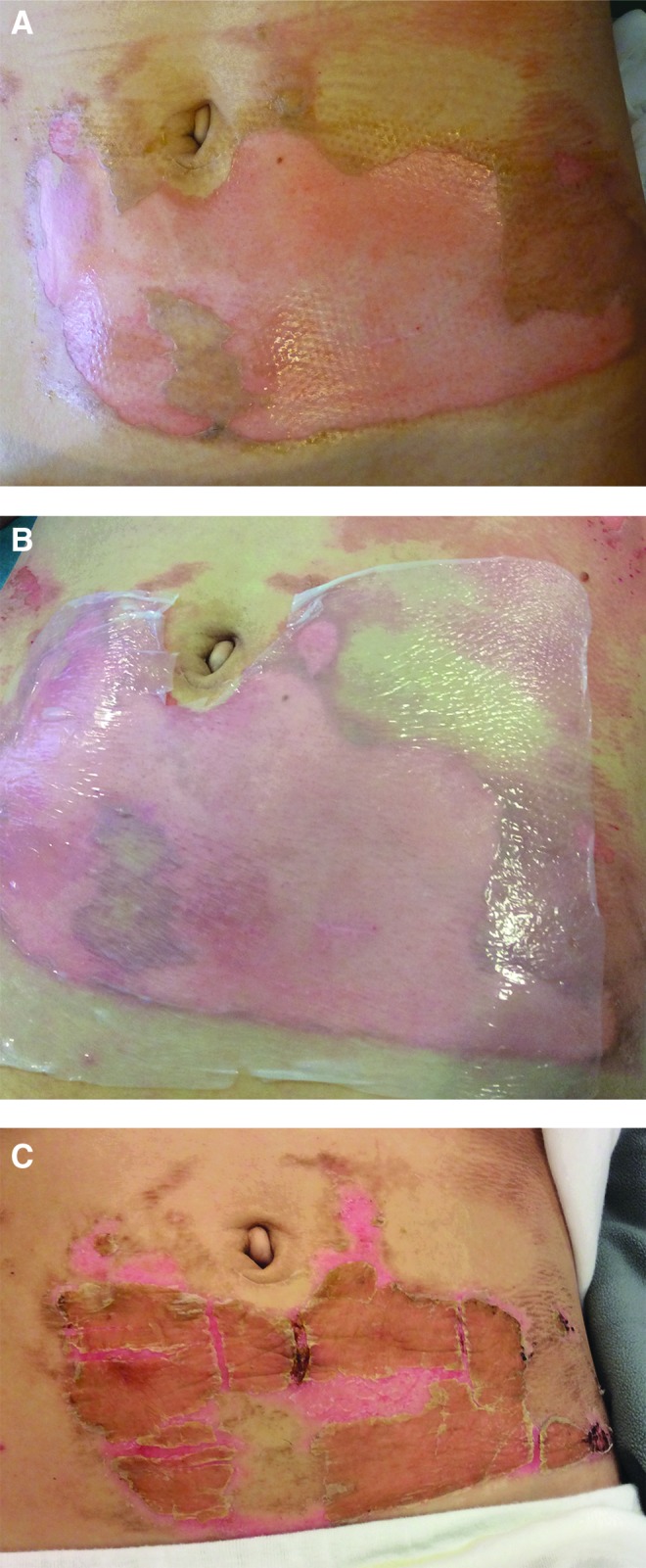
**(A)** Superficial dermal burn to the abdomen. **(B)** Biosynthetic cellulose used to cover the wound. **(C)** As healing progresses during the course of 1–2 weeks, the biosynthetic cellulose dries and peels off.

**Figure 2.**
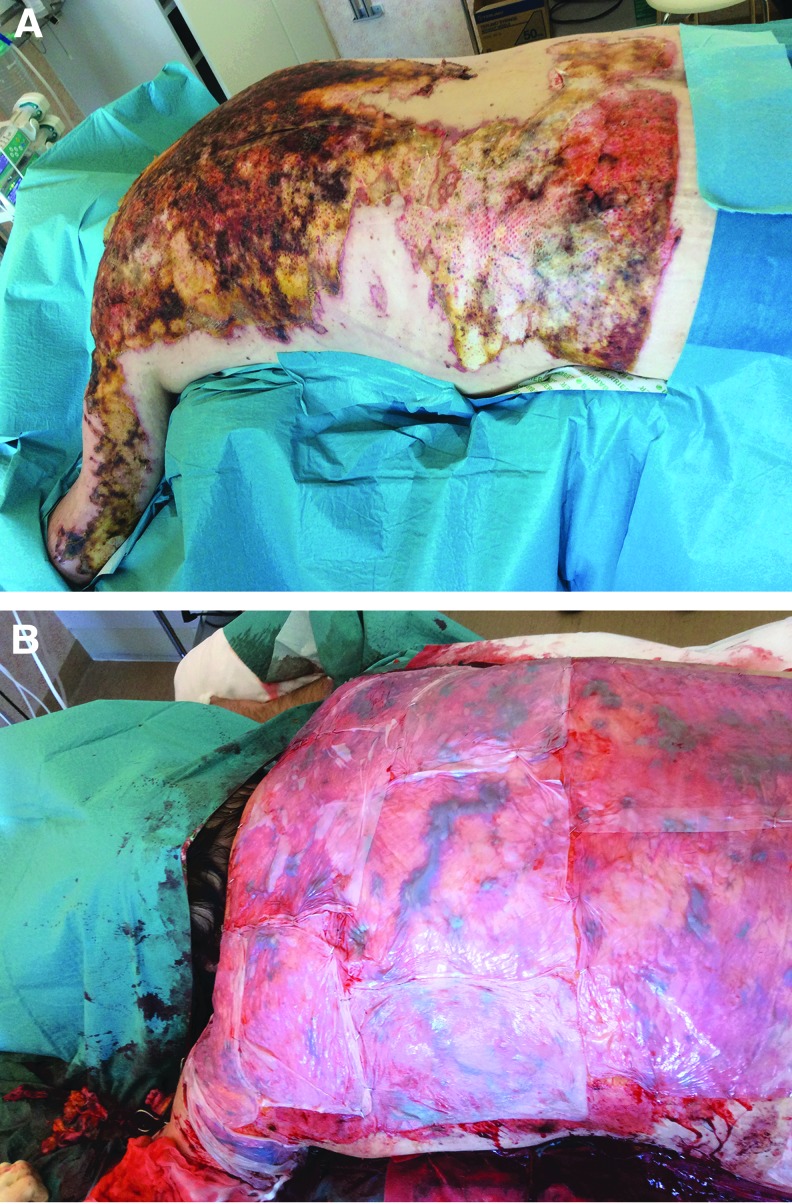
**(A)** Full-thickness burn to the back before excision. **(B)** Excised wound bed temporarily covered with biosynthetic cellulose.

Thirty-eight patients fulfilled the inclusion criteria. In 18 patients, the biosynthetic cellulose was applied on a superficial wound bed that was cleaned but not excised (SB group) (Fig. 1). In 20 of the patients, the dressing was applied on excised wounds before transplantation with autologous split thickness skin grafts (EB group) (Fig. 2). For the SB group, 6 patients were female and median age was 40 (1–89) years, and for the excised group, 4 patients were female and the median age was 56 (42–85) years. The extent of the burns as total burn surface area is stated in Table 1.

**Table 1.** Burn surface area by treatment group

	*Superficial Burns (*n* = 18)*	*Excised Burns (*n* = 20)*
TBSA%	8.6 (0.6–21.1)	25.9 (6.8–45.1)
Full thickness%	0 (0–2.3)	8 (0–26.3)
Deep dermal%	1.4 (0–11.1)	4.5 (0–21.3)
Superficial dermal%	3.0 (0–14.8)	2.3 (0–8.4)

TBSA%, percentage of total body surface area burned.

For both SB and EB groups, a vast majority of the patients (35/38) had one application of the biosynthetic cellulose only. The median number of sheets per patient was 8 (2–25), and for the two patients with the largest area covered with cellulose 30 and 31 sheets were used, respectively. Patients with SBs (SB group) reported pain relief right after application.

Eleven patients in each burn group presented positive wound swabs at treated areas within the first week after application, and in seven patients with SBs (39%; SB group), wound infections were confirmed. Of the EBs (EB group), all 11 had confirmed wound infection (61%). The most frequent bacteria found in positive wound swabs was *Staphylococcus aureus* (36% of swabs; SB and EB groups together).

The median healing time (final wound closure) for burn patients treated conservatively (SB group) was 28 (13–80) days. The median duration of hospital stay for the patients with EB wounds (EB group) was 35 days compared with 11 days for the SBs (SB group).

## Discussion

The data reported in this retrospective case series show the potential of biosynthetic cellulose (Epiprotect^®^) for acute burns. The dressing was used successfully in both SB (SB group) and EB (EB group) with no major or minor problems or adverse effects, locally or systemically.

All wounds in the conservatively treated group with superficial wounds (SB group) healed as expected, and hospital stay (11 days) is similar to that reported by Troy *et al.* in a review of 157 patients using PX in partial-thickness burns, even though time to wound closure was longer (28 days).^3^

The ease of pain when applying the cellulose has been reported by a majority of the patients with conservatively treated superficial wounds (SB group) in our study. This was a somewhat unexpected finding, and thus objective data of pain relief were not recorded prospectively, so it could not be extracted from the medical records in our material. However, this phenomenon has been reported by others,^25,26^ and it is likely to be a characteristic of the biosynthetic cellulose, possibly due to wet contact to free nerve endings in a similar manner as when putting a burnt finger in tap water to ease pain. Since no quantifiable metric was used in this case series, this finding needs to be looked further into future studies before any conclusions can be drawn.

In our experience some patients also report temporary pain relief upon wound contact in instances when PX is used. This is probably attributed to the xenograft that is wet initially either from the fluid in which it is stored (nonfrozen) or from the saline in which it is thawed before use (frozen). However, when the xenograft gets dry, this effect is less obvious. The pain relief that we have seen with the biosynthetic cellulose in our patients is in our experience more pronounced and longer lasting than that seen with xenografts, possibly due to the structure of cellulose that is likely to hold water.

In a recent study by us, we have seen that donor sites dressed with PXs exhibit unfavorable scarring after 8 years compared with donor sites dressed with polyurethane foam, although time to healing was shorter^27^ (Karlsson *et al.*, accepted). Reasons for longer time to wound closure in this material needs to be looked further into, but the results from the above mentioned study points to that the longer healing time at least should not affect long-term results and scarring.

Four patients had, according to their records, noted that the cellulose got “stiff” over joints during the first days of healing. In our experience, this is also seen in more dry dressings such as vaseline gauze or PXs, but in contrast to these dressings, the dry biosynthetic cellulose could return to its initial moist form by wetting the cellulose with water or saline as the material is highly absorbent.

We have noted that the biosynthetic cellulose is transparent initially after application and thus allows inspection of excised wounds, facilitating the detection of hematomas after excisions or early infection. Xenografts are thin but dense and totally cover the wound bed, making it impossible to inspect the wound bed without lifting the xenograft. This maneuver risks interfering with healing and should be discouraged unless there is infection or lack of healing is suspected. Therefore, the transparency of biosynthetic cellulose directly after application is said to be a way to evaluate the wound bed without having to remove the dressing.^26^ In our experience, the biosynthetic cellulose allows wound inspection on superficial wounds when healing is progressing without any infection, which decreases the need for lifting the dressing for inspection. In cases where infection causes a very wet environment or when there is a lot of oozing of wound fluid from the wound, it makes the biosynthetic cellulose a opalescent and more difficult to see through. However, under the latter circumstances the biosynthetic cellulose is still less dense and more transparent than xenograft or silicone-based foam alternatives.

It is generally accepted that bacterial colonization in burn wounds is close to a normal state after some time of treatment. To draw the line between colonization and manifest infection is not always easy and is a question for debate. We choose to define infection as positive swabs, elevated CRP, and diagnosed as clinically infected from the medical records as judged by the examiner. Based on these criteria, the infection rate seen in the studied group (39% and 61%) is higher compared to other studies. This may be explained by the differences in the criteria in diagnosis of burn wound infection, which may also affect the reported infection rate in different studies.^28,29^ This finding might of course also be a disadvantage of the material itself because it exhibits no antimicrobial activity. However, biosynthetic cellulose^26^ has been combined with zinc oxide,^30^ silver nanoparticles,^10,31,32^ or even gold^33^ *in vitro* to prevent bacterial growth, and possibly this would also exert antimicrobial properties *in vivo*. The ability of biosynthetic cellulose to be combined with antimicrobial agents could be utilized to further control infections in the future to limit the use of systemic antibiotics.

Since the biosynthetic cellulose does not exhibit some of the drawbacks that are seen with animal derived tissue, biosynthetic cellulose makes, in our experience drawn from these case series, a good alternative to PX in conservatively treated burn patients or for temporary coverage of EBs.

## Innovation

The biosynthetic cellulose used in this study is a new material for dressings. It is CE-marked, off-the-shelf product, stored in room temperature, and commercially available (Epiprotect^®^; S2Medical AB, Linköping, Sweden). It is moist and semitransparent, and it comes in 1–2 mm thick sheets (size 17 × 21 cm), that are applied directly onto the clean wound bed in the same way as PX. The difference between PX and biosynthetic cellulose is that it is free from human or animal tissue, and its semitransparancy allows monitoring the wound through the dressing initially.

Key Findings In our practice, biosynthetic cellulose (Epiprotect^®^) is a promising alternative to other dressings for SBs and a temporary cover on EBs:

- It seems to have an ability to keep a moist wound environment and thus provide the same conditions for healing as xenografts.
- It has semitransparent properties in the initial course of healing contrary to nontransparent PXs.
- It is not associated with the ethical or cultural issues, which is the case for PXs.
- It seems to provide pain relief upon application, possibly due to moist coverage of free nerve endings.

## About the Authors

All the authors have at least one of their affiliations with the National Burn Unit, Linköping, Sweden. **Matilda Karlsson, RN,** is a Registered Nurse, lecturer, and PhD-student at the National Burn Unit mainly working with advanced wound care in the burn unit. **Pia Olofsson, MD, PhD,** is a Consultant Plastic Surgeon at the National Burn Unit and a PhD. **Ingrid Steinvall, PhD,** is a Registered Nurse and PhD with special interest in statistics apart from advanced burn care at the National Burn unit. **Folke Sjöberg, MD, PhD,** is a Consultant Anesthesiologist, Professor of Burn Care, and Director of the National Burn Unit. **Johan Thorfinn, MD, PhD,** is a Consultant Plastic Surgeon at the National Burn Unit and Associate Professor of Plastic Surgery. **Moustafa Elmasry, MD, PhD,** is a Specialist of Plastic Surgery and a PhD at the National Burn Unit.

## Abbreviations and Acronyms

CRP: C-reactive protein
EB: excised burns
PX: porcine xenograft
SB: superficial burn
